# Febrifugine dihydrochloride restricts porcine epidemic diarrhea virus replication by modulating the IGF1R-driven PI3K/AKT-apoptosis axis

**DOI:** 10.1128/jvi.00117-26

**Published:** 2026-04-15

**Authors:** Ping Yan, Zin Oo Kyaw, Xiaobing Wang, Nan Li, Yulan Xu, Song Gao, Changchao Huan

**Affiliations:** 1 Institute of Agricultural Science and Technology Development, College of Veterinary Medicine, Yangzhou University 38043, Yangzhou, China; 2 Jiangsu Co-Innovation Center for Prevention and Control of Important Animal Infectious Diseases and Zoonoses 614704 , Yangzhou, China; 3 Key Laboratory of Avian Bioproduct Development, Ministry of Agriculture and Rural Affairs 38043, Yangzhou, China; University of Kentucky College of Medicine, Lexington, Kentucky, USA

**Keywords:** febrifugine dihydrochloride, broad-spectrum antiviral activity, PEDV, IGF1R, PI3K/AKT, apoptosis

## Abstract

Porcine epidemic diarrhea (PED) is an acute and highly contagious enteric disease caused by porcine epidemic diarrhea virus (PEDV). The disease poses a significant threat to global swine production, underscoring the urgent need for effective antiviral therapeutics. In this study, we investigated the antiviral activity and underlying mechanism of febrifugine dihydrochloride (FFG), a quinazolinone alkaloid derived from *Dichroa febrifuga*. Although traditionally recognized for its antimalarial properties, the antiviral potential of FFG has remained largely unexplored. Our findings demonstrated that FFG exhibited strong and dose-dependent antiviral activity against PEDV, with its action targeting viral replication. Moreover, FFG displayed broad-spectrum antiviral activity against multiple viruses. Network pharmacology analysis combined with experimental validation identified IGF1R as a key host target of FFG. Mechanistically, FFG completely blocked the activation of the PI3K/AKT pathway induced by various viruses, establishing the host signaling axis as a critical node for its antiviral activity. We subsequently demonstrated that FFG inhibited PEDV replication by inducing apoptosis through the downstream cascades of the PI3K/AKT pathway. Notably, FFG’s antiviral mechanism was effective against multiple viruses, highlighting its role in targeting conserved host antiviral mechanisms. Collectively, these findings identify FFG as a promising host-directed antiviral candidate. By targeting the IGF1R-PI3K/AKT-apoptosis axis, FFG modulates critical host signaling pathways to restrict viral replication, providing valuable mechanistic insight and new therapeutic targets for the development of antiviral agents.

**IMPORTANCE:**

Porcine epidemic diarrhea virus (PEDV) remains one of the most devastating pathogens threatening the global swine industry, yet effective antiviral treatments are still lacking. In this study, we identified FFG as a promising broad-spectrum antiviral agent with dramatic effects on PEDV. The PI3K/AKT signaling pathway is a central phosphorylation cascade involved in the regulation of diverse cellular processes and frequently exploited by viruses to facilitate infection. Our results demonstrated that FFG suppressed PEDV replication through PI3K/AKT-dependent modulation of apoptosis, and the regulatory effect was mediated by targeting host receptor IGF1R. Collectively, these findings provide insight into host-virus interactions and highlight the IGF1R-PI3K/AKT-apoptosis axis as a promising target for antiviral therapy.

## INTRODUCTION

Porcine epidemic diarrhea (PED) is a highly contagious disease caused by porcine epidemic diarrhea virus (PEDV), a member of the genus Alphacoronavirus (1). First reported in the United Kingdom in 1971, PEDV was confirmed to be the etiological agent in 1978; however, its initially relatively low mortality rate limited global attention (2). Since 2010, highly pathogenic variants of PEDV have progressively spread, resulting in extremely high mortality rates in neonatal piglets (3, 4). Despite the development of multiple vaccines, effective control of PEDV remains challenging due to the lack of durable mucosal immunity, particularly against heterologous strains, and the viral ability to evade host immune responses (5, 6). These challenges underscore the urgency of developing effective antiviral drugs to treat PEDV infection.

Natural products have long been recognized as a rich source of antiviral compounds, primarily due to their diverse chemical scaffolds and multitarget mechanisms of action. This inherent diversity allows natural products to interact with multiple viral targets, thereby offering a broad spectrum of antiviral activities (7). Febrifugine dihydrochloride (FFG) is the dihydrochloride form of febrifugine, developed to improve its solubility and stability, and belongs to the quinazolinone alkaloid extracted from the roots and leaves of *Dichroa febrifuga* with significant antimalarial activity (8–10). Recent studies have demonstrated that FFG exhibits significant pharmacological activities, including antimalarial, anti-inflammatory, and antitumor effects (11–13). The underlying mechanisms primarily involve inhibition of tumor cell proliferation and induction of apoptosis (14). Ongoing studies are exploring the antiviral potential of FFG, with the aim of expanding its therapeutic applications.

As is known, apoptosis, or programmed cell death, is a critical host defense mechanism that limits viral spread by eliminating infected cells (15). It represents an essential antiviral response activated by the host to hinder virus replication and spread, thus protecting other cells and tissues (16). Moreover, the apoptosis in infected cells facilitates antigen presentation and immune cell recruitment, thereby helping to resolve the infection (17). Zhang et al. found that rotenone, a natural isoflavonoid compound usually obtained from the roots and stems of leguminous plants, suppressed RSV infection by modulating virus-induced apoptosis (18). Similarly, chrysin, a natural flavone usually obtained from honey and propolis, reduced influenza virus infection in the upper respiratory tract by mitochondria-dependent apoptosis (19). However, many viruses have evolved sophisticated strategies to manipulate apoptotic pathways to favor their replication (20, 21). The PEDV Nsp9 interacted with the host factor H2BE and promoted viral replication by modulating endoplasmic reticulum stress to inhibit apoptosis (22). Similarly, Si et al. showed that PEDV ORF3 facilitated its replication by inhibiting apoptosis (23). As reported by Qiao and colleagues, matrine, a quinolizidine alkaloid usually obtained from plants of the genus *Sophora*, exerted its antiviral activity by regulating apoptosis via direct targeting of spike proteins (24). Given that multiple PEDV proteins are closely associated with apoptosis regulation, it is evident that apoptosis is intricately linked to PEDV replication.

Building on these pharmacological insights, the study aimed to systematically evaluate the antiviral potential of quinazolinone alkaloid derived from *Dichroa febrifuga* against PEDV infection, as illustrated in Fig. S1. Among the compounds tested, FFG exhibited significant antiviral activity, and its underlying antiviral mechanism was elucidated. These findings provide new insights into the development of plant-derived antiviral candidates.

## RESULTS

### Screening of *Dichroa febrifuga*-derived quinazolinone alkaloids with antiviral activity

**Fig 1:**
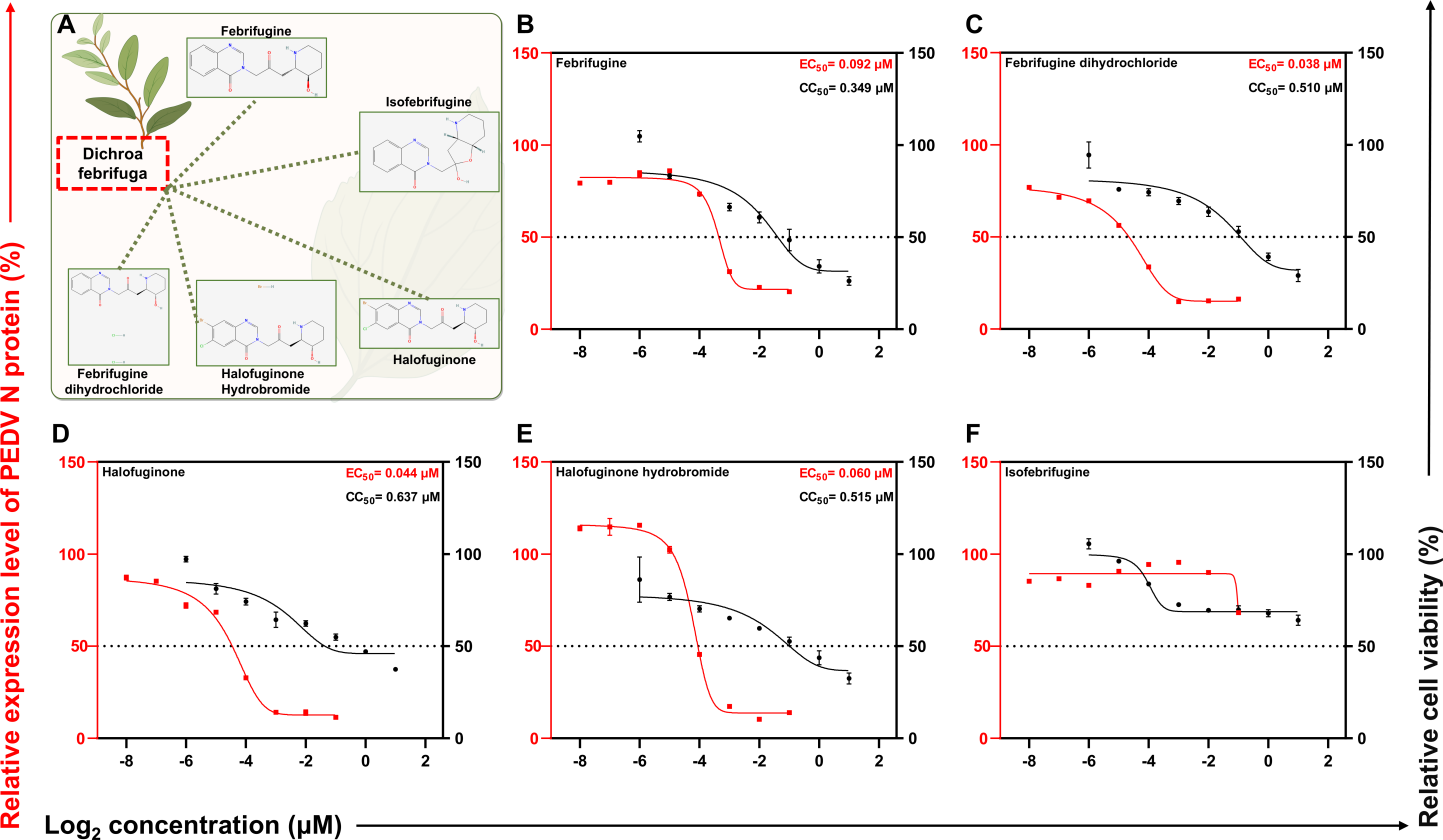
Antiviral screening of quinazolinone alkaloids from *Dichroa febrifuga* against PEDV. (**A**) Chemical structures of five representative compounds: febrifugine, febrifugine dihydrochloride, halofuginone, halofuginone hydrobromide, and isofebrifugine. (**B–F**) Dose-response antiviral activity and cytotoxicity of the five compounds. EC_50_ (red) and CC_50_ (black) curves of each compound were established in Vero cells. Mean values ± SD were shown (*n* = 3 biologically independent samples).

To systematically assess the antiviral activity of *Dichroa febrifuga*-derived quinazolinone alkaloids, we selected five representative compounds, including febrifugine, febrifugine dihydrochloride (FFG), halofuginone, halofuginone hydrobromide, and isofebrifugine (Fig. 1A), and evaluated their ability to inhibit PEDV infection in Vero cells. Antiviral activity was initially assessed with the detection of N protein level by Western blotting, and cell viability was evaluated via CCK-8 assays, followed by quantitative determination of EC₅₀, CC₅₀, and selectivity index (SI = CC₅₀/EC₅₀). Dose-response analyses revealed that five compounds exhibited measurable antiviral activity, although with varying degrees of potency and cytotoxicity (Fig. 1B through F). Among them, FFG exhibited the strongest inhibition against PEDV infection, with EC₅₀ of 0.038 μM and CC₅₀ of 0.510 μM, yielding a favorable SI of 13.4. Febrifugine, halofuginone, and halofuginone hydrobromide also displayed submicromolar antiviral activity with EC₅₀ of 0.092 μM, 0.044 μM, and 0.060 μM, respectively, corresponding to SI of 3.80 (febrifugine), 14.5 (halofuginone), and 8.6 (halofuginone hydrobromide). In contrast, isofebrifugine demonstrated only marginal antiviral activity at 2 μM.

Taken together, these quinazolinone alkaloids from *Dichroa febrifuga* represent a structurally diverse class with significant anti-PEDV potential. FFG exhibited submicromolar efficacy with an SI of 13.4, suggesting its potential as a lead compound for further mechanistic investigation.

### FFG suppresses PEDV infection

**Fig 2:**
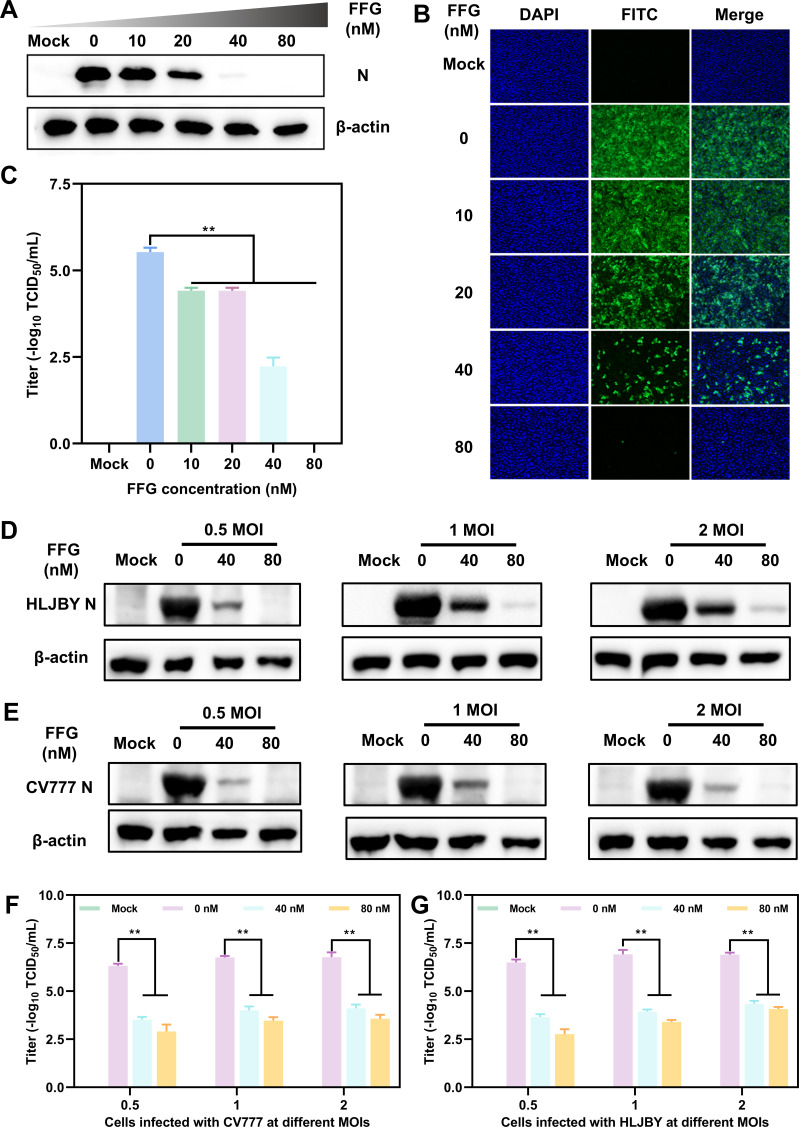
The antiviral effect of FFG against PEDV. Vero cells were pre-treated with increasing concentrations of FFG for 1 h and subsequently infected with PEDV strains HLJBY or CV777 at different MOIs in the presence of FFG. After infection, the cells were co-cultured with FFG for 24 hpi, and then the samples were subsequently collected to assess viral infection. (**A**) The viral protein levels were assessed via Western blotting. (**B**) Internalized viruses were detected by IFA. (**C**) Viral titers in cell supernatants were determined by TCID_50_. (**D** and **E**) N protein levels of HLJBY and CV777 at various MOIs, respectively. (**F** and **G**) Viral titers of HLJBY and CV777 at various MOIs, respectively. The mean values ± SD were shown (*n* = 3). Statistical significance: *P < 0.05; **P < 0.01; ns, not significant. Typical images were presented. The subsequent data analysis and significance annotations remained consistent.

To further validate the antiviral efficacy of FFG against PEDV, Vero cells were treated with increasing concentrations of FFG and subsequently infected with PEDV/HLJBY. The Western blotting results showed a dose-dependent reduction in PEDV N protein accumulation, with expression levels decreasing by 51%, 98%, and 99% at 20, 40, and 80 nM, respectively, compared with FFG untreated controls (Fig. 2A). Consistently, IFA further confirmed the inhibitory effect of FFG via observing PEDV-positive cells (Fig. 2B). In agreement with these observations, TCID₅₀ assays demonstrated that FFG markedly reduced viral titers, with infectivity decreased by 3.3 logs and 5.3 logs at 40 and 80 nM, respectively (Fig. 2C). To determine whether the antiviral activity of FFG was influenced by the MOIs of PEDV infection or viral genotype, PEDV strains HLJBY and CV777 were evaluated at MOIs of 0.5, 1, and 2. The Western blotting results revealed that FFG retained significant antiviral activity against both the HLJBY (Fig. 2D) and CV777 (Fig. 2E) across different MOIs. Quantification of viral titers in culture supernatants confirmed that FFG treatment resulted in consistent decreases of 2.2–3.7 logs across all MOIs and viral strains (Fig. 2F and G).

Together, these results demonstrated that FFG exerted a potent and concentration-dependent antiviral effect against PEDV, effectively suppressing both viral protein synthesis and progeny virus production across varying infection intensities and viral genotypes.

### FFG demonstrates broad-spectrum antiviral activity

**Fig 3:**
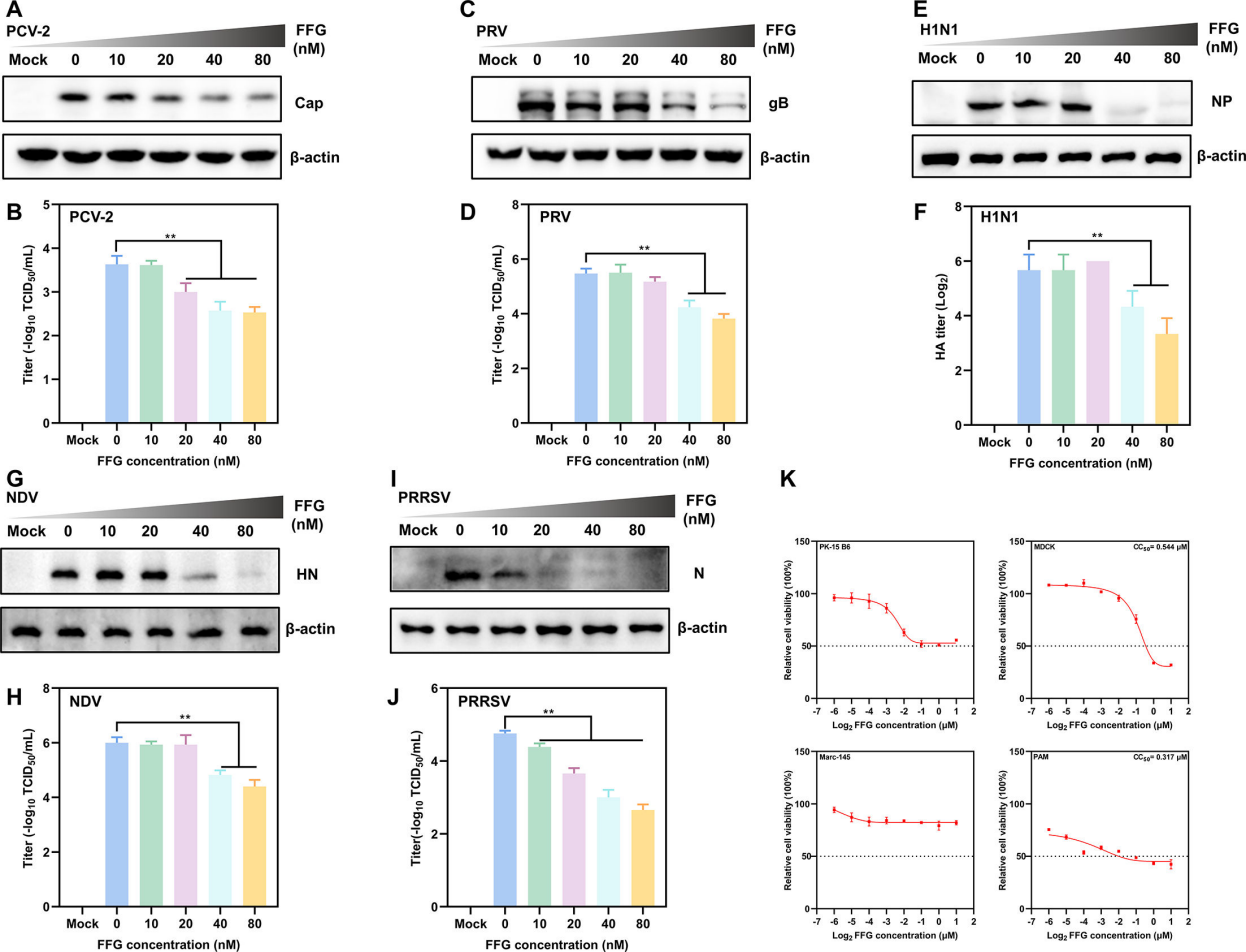
Broad-spectrum antiviral activity of FFG. Viral protein levels and titers were assessed via Western blotting and TCID_50_ assays in cells infected with PCV-2, PRV, H1N1, NDV, or PRRSV and treated with FFG for 24 hpi. (**A**, **C**, **E**, **G**, and **I**) Expression levels of viral proteins by Western blotting, respectively. (**B**, **D**, **F**, **H**, and **J**) Viral titers by TCID_50_ or hemagglutination assays, respectively. (**K**) The cell viability of FFG in PK-15 B6, MDCK, Marc-145, and PAMs cells was determined by CCK-8 assays after 24-h treatment. The mean values ± SD were shown (*n* = 3).

Given the potent inhibitory effect of FFG against PEDV, we next investigated whether its antiviral efficacy extended to other clinically and economically relevant viral pathogens. To this end, five representative viruses from distinct viral families were selected, including porcine circovirus type 2 (PCV-2), pseudorabies virus (PRV), influenza A virus (H1N1), Newcastle disease virus (NDV), and porcine reproductive and respiratory syndrome virus (PRRSV). By treating cells with FFG at increasing concentrations, viral protein expression levels and infectious viral titers were subsequently assessed to evaluate its broad-spectrum antiviral activity. As shown in Fig. 3, FFG treatment resulted in a concentration-dependent reduction in viral replication across all tested viruses. The Western blotting analysis revealed that FFG markedly suppressed viral protein expression, with PCV-2 Cap protein levels reduced by 53% at 80 nM (Fig. 3A). For PRV, H1N1, NDV, and PRRSV, viral protein levels were reduced by 70%–99% at the same concentration compared with untreated controls (Fig. 3C, E, G and I). Consistent with these findings, TCID₅₀ assays revealed significant decreases in infectious viral titers, ranging from 1.6 to 2.2 logs for PCV-2, PRV, NDV, and PRRSV (Fig. 3B, D, H and J). In the case of H1N1, hemagglutination assays showed a greater than 90% reduction in HA titers following FFG treatment (Fig. 3F). The cytotoxicity assays were performed in the corresponding host cell lines used for each viral infection model, including Vero cells (PEDV and NDV), PK-15 B6 (PRV and PCV-2), MDCK (H1N1), Marc-145 (PRRSV), and PAMs. The results demonstrated that FFG exhibited cell type-dependent cytotoxicity, with CC₅₀ of 0.510 μM for Vero (Fig. 1C), 0.544 μM for MDCK, and 0.317 μM for PAMs (Fig. 3K). Furthermore, EC₅₀ values were derived from dose-response curves generated using densitometric quantification of viral protein bands, yielding EC₅₀ values of 0.058 μM (PCV-2), 0.037 μM (PRV), 0.036 μM (H1N1), 0.042 μM (NDV), and 0.017 μM (PRRSV). Based on these data, the SI of FFG was 15.11 against H1N1 and 12.14 against NDV. Notably, the EC₅₀ values of FFG against all tested viruses were within the micromolar range and remained below the corresponding CC₅₀ values, suggesting that antiviral efficacy was achieved at concentrations not associated with overt cytotoxicity under the experimental conditions.

### FFG does not affect PEDV attachment, entry, or virions

By referring to previous studies in our laboratory (25), we next sought to pinpoint the specific stage of the PEDV life cycle at which FFG exerts its antiviral effects, including viral attachment, entry, replication, and progeny release. Vero cells were treated with increasing concentrations of FFG (0, 10, 20, 40, and 80 nM) and infected with PEDV for defined periods, respectively.

**Fig 4:**
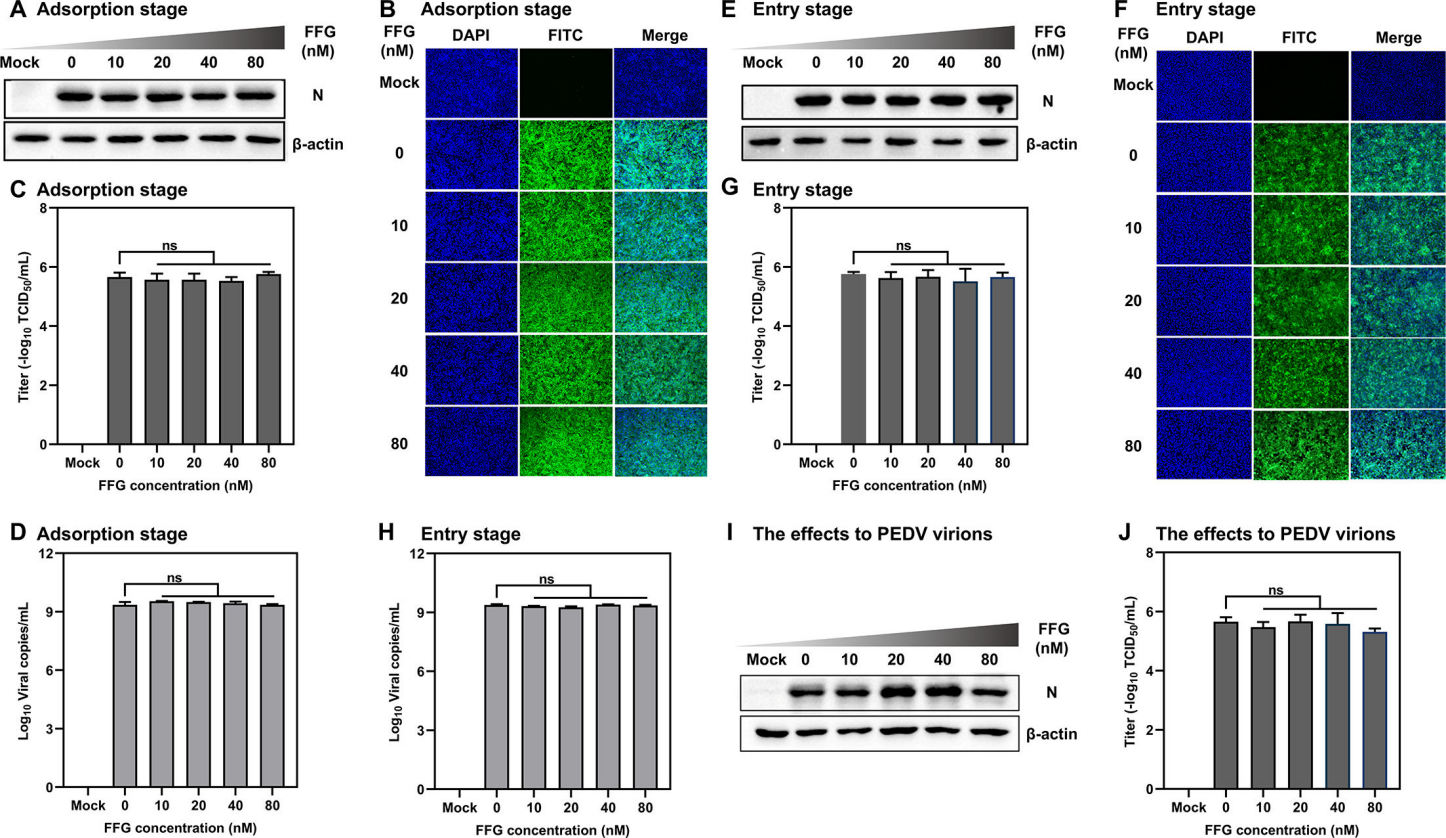
FFG did not affect viral adsorption, entry, or viral particle integrity. For the adsorption assay, Vero cells were infected with 0.1 MOI PEDV/HLJBY at 4°C in the presence of different concentrations of FFG for 1 h, and then the cells were cultured without FFG for 24 hpi. (**A**) PEDV N proteins were detected via Western blotting. (**B**) The internalized virus was detected by IFA. (**C**) The supernatant viral titers were determined by TCID_50_. (**D**) At 1 hpi, PEDV/HLJBY copy numbers were quantified by qRT-PCR. For the entry assay, Vero cells were infected with 0.1 MOI PEDV/HLJBY at 4°C for 1 h, and then incubated with various concentrations of FFG for 1 h at 37°C, followed by washing off the extracellular virus and incubation without FFG for 24 hpi. (**E**, **F**, and **G**) PEDV N protein levels, internalized virus, and viral titers, respectively. (**H**) At 2 hpi, intracellular viral copy numbers were quantified by qRT-PCR. To assess viral particle integrity, PEDV/HLJBY virions were incubated with increasing concentrations of FFG for 1 h and subsequently used to infect Vero cells for 24 hpi. (**I** and **J**) PEDV N protein levels and viral titers, respectively. The mean values ± SD were shown (*n* = 3), and typical images were presented.

To first evaluate whether FFG interfered with PEDV attachment to host cells, Vero cells were pretreated with DMEM containing various concentrations of FFG and inoculated with PEDV/HLJBY at 4°C for 1 h. Following incubation, unbound virions were removed by washing with pre-chilled PBS, and cells were incubated in 2% FBS DMEM at 37°C for 24 h post-infection (hpi). The Western blotting results showed that FFG treatment did not alter PEDV N protein levels (Fig. 4A). Consistently, analyses of internalized virus detected by IFA (Fig. 4B), viral titers measured by TCID₅₀ (Fig. 4C), and viral copy number quantified by qRT-PCR (Fig. 4D) showed no significant difference between FFG-treated and -untreated groups. These results indicated that FFG did not affect the adsorption stage of PEDV.

Next, we evaluated the effect of FFG on the viral entry stage. Vero cells were incubated with PEDV/HLJBY at 4°C for 1 h to permit attachment. After washing with PBS to remove unbound virions, cells were treated with 2% FBS DMEM containing different concentrations of FFG and incubated at 37°C for 1 h to allow viral internalization. Subsequently, cells were washed with citric acid solution to remove residual surface-bound viruses, then cultured in FFG-free 2% FBS DMEM for 24 hpi. Consistent with the results observed during the attachment stage, FFG showed no effect on PEDV entry into host cells (Fig. 4E–H). To determine whether FFG directly affects viral integrity or infectivity, PEDV/HLJBY virions were preincubated with FFG for 1 h before infection of Vero cells. As shown in Fig. 4I and J, Western blotting and TCID₅₀ results revealed that FFG did not impair viral infectivity.

### FFG targets the PEDV replication stage for inhibiting viral infection

**Fig 5:**
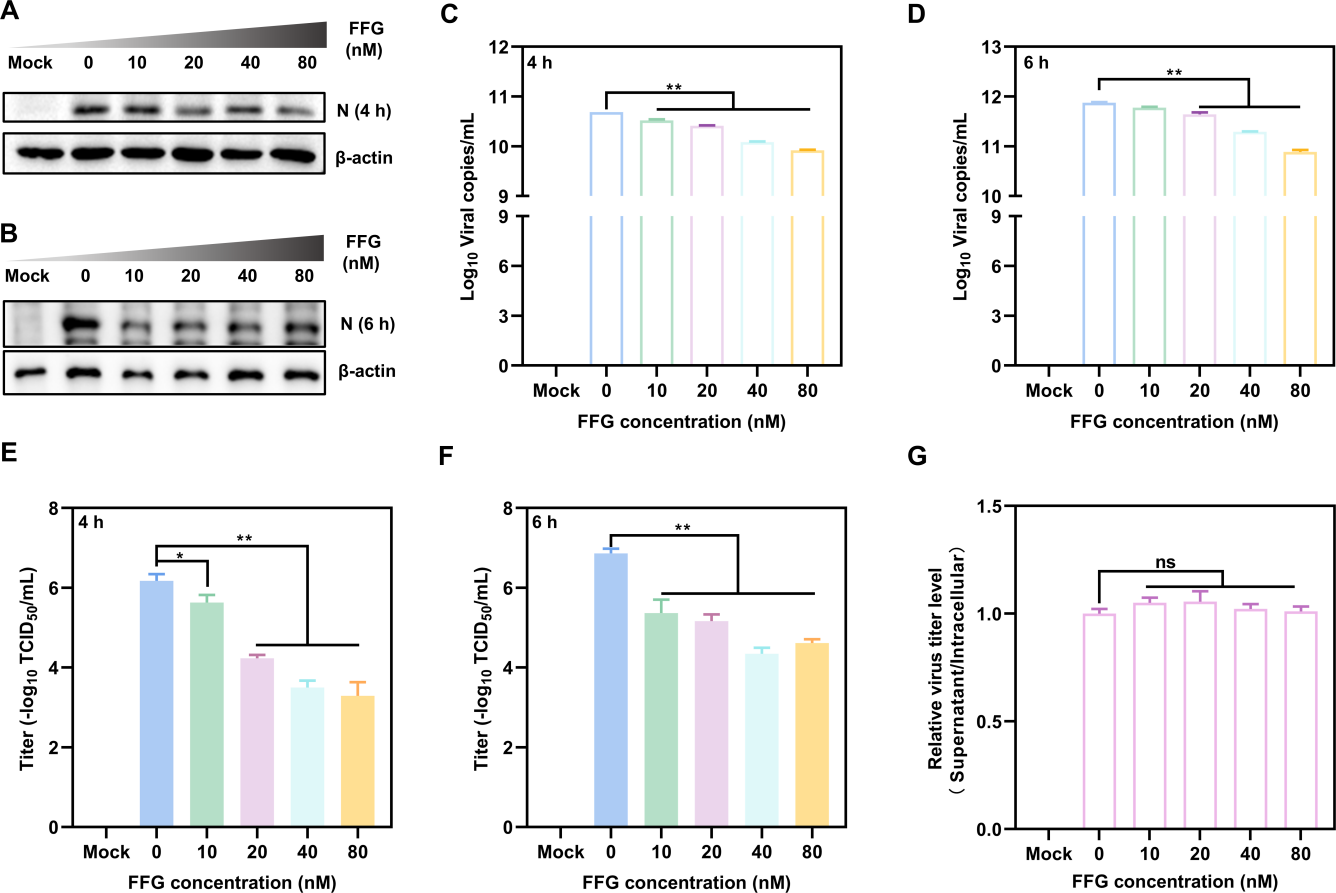
FFG exerted antiviral activity by targeting the viral replication stage. Vero cells were infected with 1 MOI PEDV at 37°C for 1 h and subsequently incubated with increasing concentrations of FFG for 4 or 6 hpi. (**A** and **B**) PEDV N protein levels at 4 and 6 hpi, respectively. (**C** and **D**) Intracellular viral copy numbers at 4 and 6 hpi, respectively. (**E** and **F**) Viral titers at 4 and 6 hpi, respectively. To assess the effect of FFG on viral release, Vero cells were infected with 0.1 MOI PEDV/HLJBY for 1 h, and then incubated in 2% FBS DMEM containing different concentrations of FFG. At 24 hpi, cell supernatants were collected, and cells were resuspended in 1 mL DMEM and subjected to repeated freeze-thaw cycles to release intracellular virions. Viral titers were determined for both fractions by TCID_50_, and the ratio of supernatant to intracellular TCID₅₀ values was calculated to evaluate viral release efficiency. (**G**) Ratio of viral titers in culture supernatants to intracellular fractions. The mean values ± SD were shown (*n* = 3).

To determine whether FFG interfered with the replication stage of PEDV, Vero cells were infected with PEDV/HLJBY and subsequently treated with 2% FBS DMEM containing various concentrations of FFG and incubated for 4 or 6 h. The Western blotting results showed a dose-dependent reduction in PEDV N protein levels following FFG treatment. Specifically, N protein levels were reduced by 10%–50% at concentrations ranging from 10 to 80 nM at 4 hpi, with a comparable reduction range of 15%–70% observed at 6 hpi, relative to the untreated controls (Fig. 5A and B). Consistently, qRT-PCR analysis showed that FFG markedly inhibited viral genome replication, reducing PEDV copy numbers by 14%–60% at 4 hpi and by 10%–90% at 6 hpi (Fig. 5C and D). Meanwhile, viral titers were significantly decreased in both a concentration- and time-dependent manner, with FFG treatment reducing titers by 2.8 logs at 4 hpi, and by 2.5 logs at 6 hpi (Fig. 5E and F). In addition, co-incubation of PEDV-infected cells with FFG had no effect on viral titers during the virus release stage, as indicated by TCID_50_ (Fig. 5G).

Collectively, these findings demonstrated that FFG potently suppressed PEDV replication in a time- and dose-dependent manner, highlighting the replication stage as the primary target of its antiviral activity.

### FFG inhibits PI3K/AKT pathway activation to suppress viral infection

**Fig 6:**
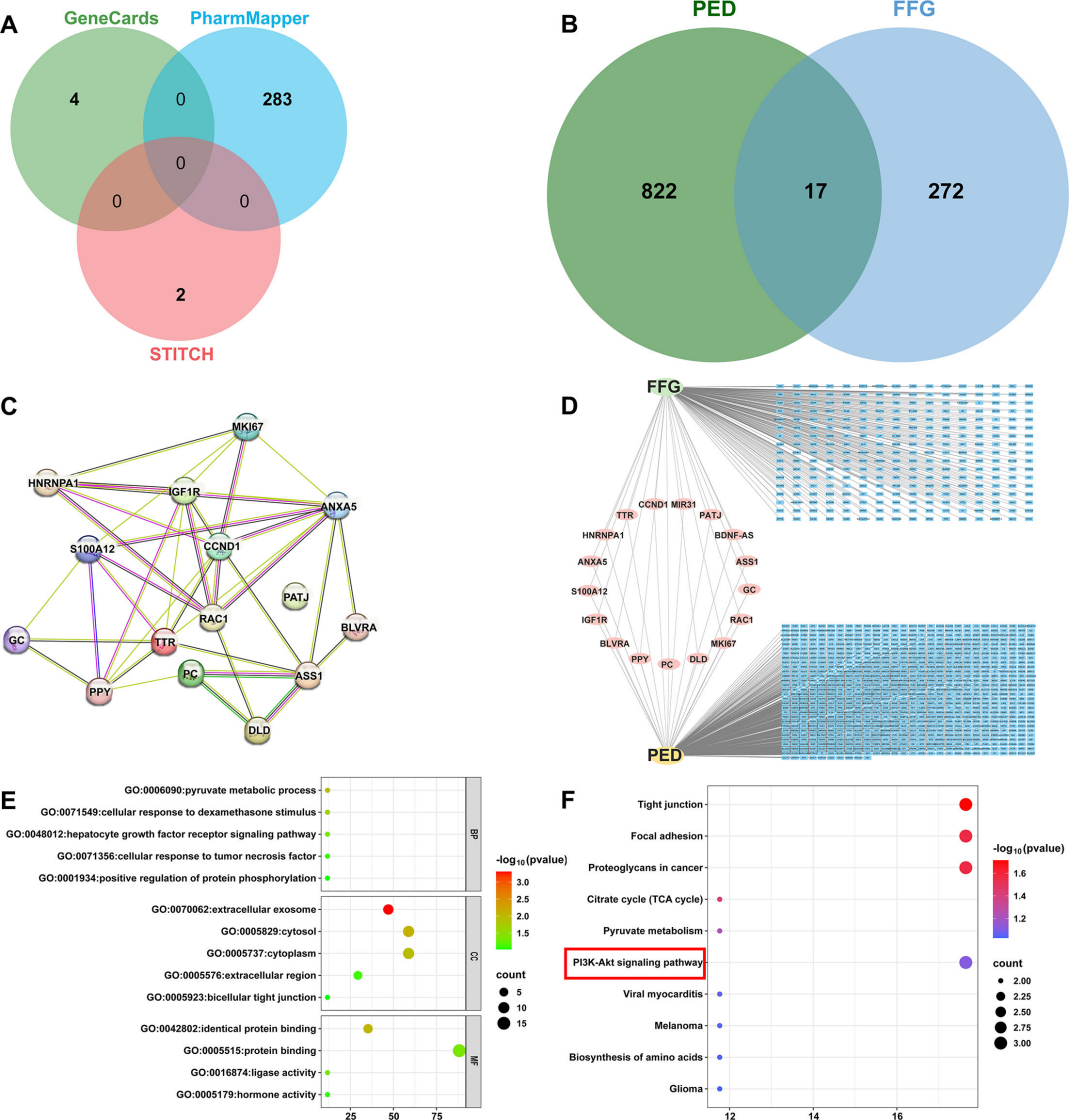
Network establishment and analysis of FFG-regulated PEDV infection. (**A**) Potential targets of FFG from different databases. (**B**) Venn diagram of the common targets between FFG and PED. (**C**) PPI network analysis. (**D**) The drug-target-disease visualization network. (**E** and **F**) GO function and KEGG pathway enrichment analysis.

Network pharmacology is a promising approach to unraveling the pharmacological mechanisms of drugs (26, 27). To further investigate the antiviral mechanism of FFG, we explored the critical targets and pathways regulated by FFG during viral infection through network pharmacology strategy, as shown in Fig. S1. Network pharmacological analyses showed that potential FFG-associated targets were predicted using the GeneCards, PharmMapper, and STITCH databases, and their intersection yielded 289 overlapping candidate targets (Fig. 6A). Meanwhile, a total of 822 PED-related targets were identified from GeneCards. Subsequently, the Venn diagram displayed 17 common targets of FFG and PED (Fig. 6B), suggesting that these proteins may play essential roles in mediating FFG’s antiviral effects. To further explore potential protein-protein interaction (PPI) among these 17 targets, PPI analysis was conducted. Upon uploading this gene data to the STRING database, a PPI network was generated, which consisted of 15 nodes, 38 connections, with an average node degree of 5.07 (Fig. 6C). The drug-target-disease network was constructed and visualized using Cytoscape software, illustrating the target genes involved in FFG-mediated regulation of PEDV infection (Fig. 6D). Functional enrichment analyses were then conducted using the DAVID online tool to assess GO terms and KEGG pathways associated with the 17 common targets. Figure 6E showed the top five biological processes (BP), cellular components (CC), and molecular functions (MF). The regulation of growth factor receptor signaling pathways and the pyruvate metabolic process was vital in biological processes. The main cellular components were the cytoplasm and the extracellular region. Besides, the molecular functions were mainly concentrated on protein binding and ligase activity. Furthermore, Fig. 6F showed the top 10 pathways, including the PI3K/AKT signaling pathway, citrate cycle (TCA cycle), and biosynthesis of amino acids.

**Fig 7:**
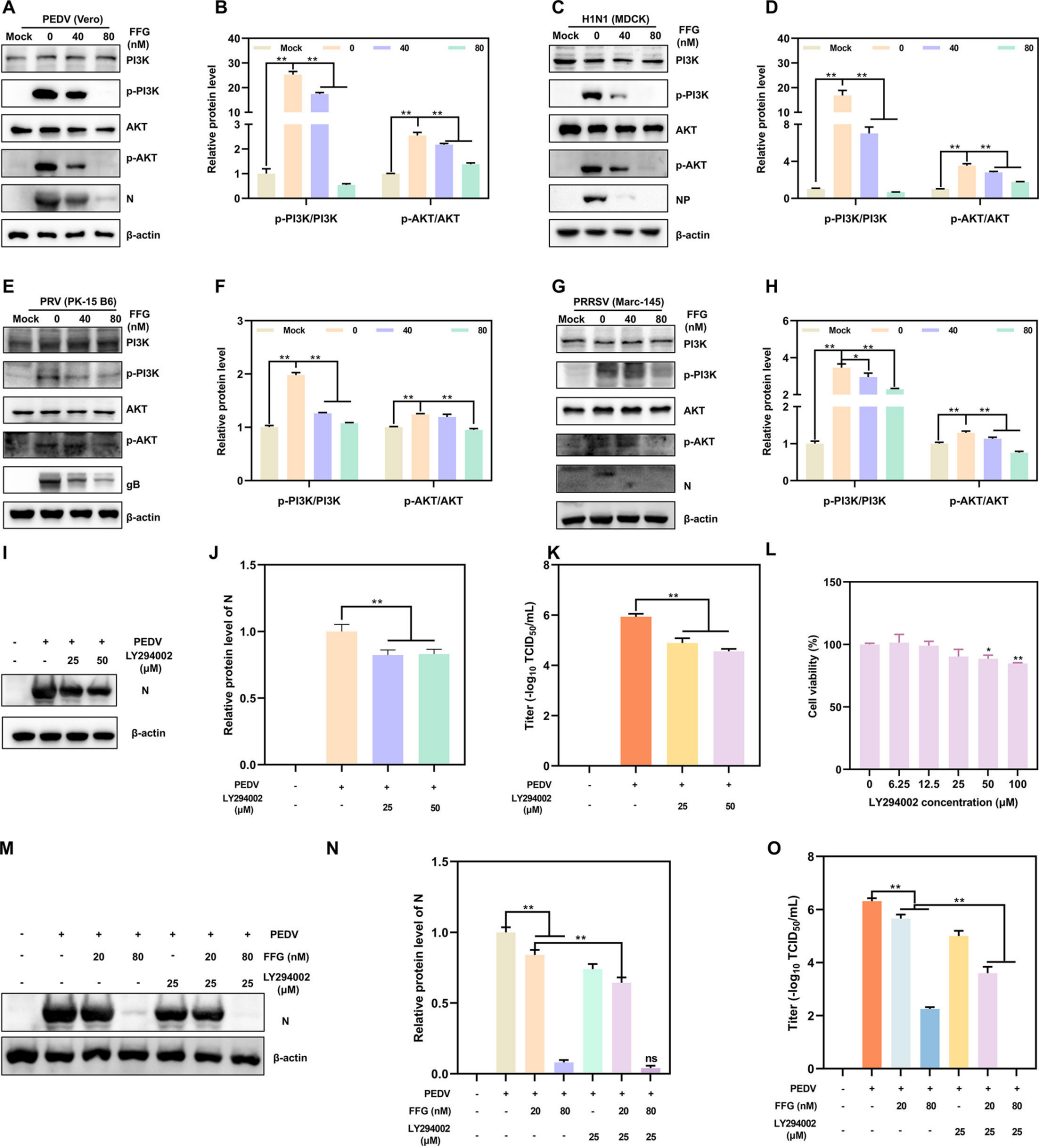
FFG blocked PI3K/AKT activation in various viruses to exert its antiviral effects. (A–H) FFG inhibited the activation of the PI3K/AKT pathway in cells infected with PEDV, H1N1, PRV, and PRRSV. Cells were infected with various viruses (0.1 MOI PEDV, 1 MOI H1N1, 0.1 MOI PRV, and 0.1 MOI PRRSV) and treated with different concentrations of FFG for 24 hpi. Cell lysates were then analyzed by Western blotting for the expression of PI3K, p-PI3K, AKT, p-AKT, and viral proteins. (**A through H**) Representative immunoblots and quantitative analysis of the ratios of p-PI3K/PI3K and p-AKT/AKT. PEDV-infected Vero cells were treated with LY294002 (PI3K-specific inhibitor) for 24 hpi, followed by the analysis of PEDV N protein expression (**I**), quantification of N protein levels (**J**), and supernatant viral titers (**K**). (**L**) The cell viability of LY294002 in Vero cells. PEDV-infected Vero cells were treated with FFG (20 or 80 nM) in the presence of 25 μM LY294002 for 24 hpi. PEDV N protein expression (**M**), quantitative analysis of N protein levels (**N**), and viral titers (**O**) were subsequently determined.

Studies have reported that the PI3K/AKT pathway plays a pivotal role in regulating virus-host interactions (28, 29). To determine whether the antiviral activity of FFG was mediated through the PI3K/AKT pathway, we first assessed the pathway activation in PEDV-infected Vero cells. PEDV infection markedly enhanced PI3K and AKT phosphorylation, whereas FFG treatment effectively suppressed the activation in a dose-dependent manner (Fig. 7A). Quantification confirmed significant reductions in p-PI3K/PI3K and p-AKT/AKT ratios at 40 and 80 nM (Fig. 7B), indicating that FFG reversed PEDV-induced PI3K/AKT activation. To evaluate whether this regulatory effect extends to other viruses, the PI3K/AKT pathway was examined in cells infected with H1N1, PRV, and PRRSV. Consistently, FFG prominently inhibited virus-induced PI3K and AKT phosphorylation in all tested models, including H1N1 (Fig. 7C and D), PRV (Fig. 7E and F), and PRRSV (Fig. 7G and H). Correspondingly, viral protein levels, including PEDV N, H1N1 NP, PRV gB, and PRRSV N, were substantially reduced following FFG treatment, confirming that suppression of the PI3K/AKT signaling cascade was closely associated with FFG’s broad-spectrum antiviral activity. To further confirm the role of the PI3K/AKT pathway in PEDV replication, PEDV-infected cells were treated with the PI3K inhibitor LY294002. As expected, LY294002 significantly reduced N protein levels (Fig. 7I and J) and viral titers (Fig. 7K), confirming that inhibition of the PI3K/AKT pathway activation restricted PEDV infection. Meanwhile, LY294002 did not significantly affect cell viability at the concentration used in the study via CCK-8 assays (Fig. 7L). Importantly, co-treatment with FFG and LY294002 further enhanced the antiviral effect of FFG, with the combination of 80 nM FFG and LY294002 nearly abolishing N protein expression (Fig. 7M and N). Consistently, viral titers were further reduced upon combined treatment. Notably, co-treatment with 80 nM FFG and LY294002 decreased viral titers from 2.25 logs to undetectable levels (Fig. 7O).

Collectively, these results indicated that FFG inhibited viral infections by blocking PI3K/AKT pathway activation, thereby interfering with cellular processes for viral propagation.

### FFG inhibits the PI3K/AKT pathway by targeting IGF1R, modulating the downstream apoptotic pathway to exert antiviral effects

**Fig 8:**
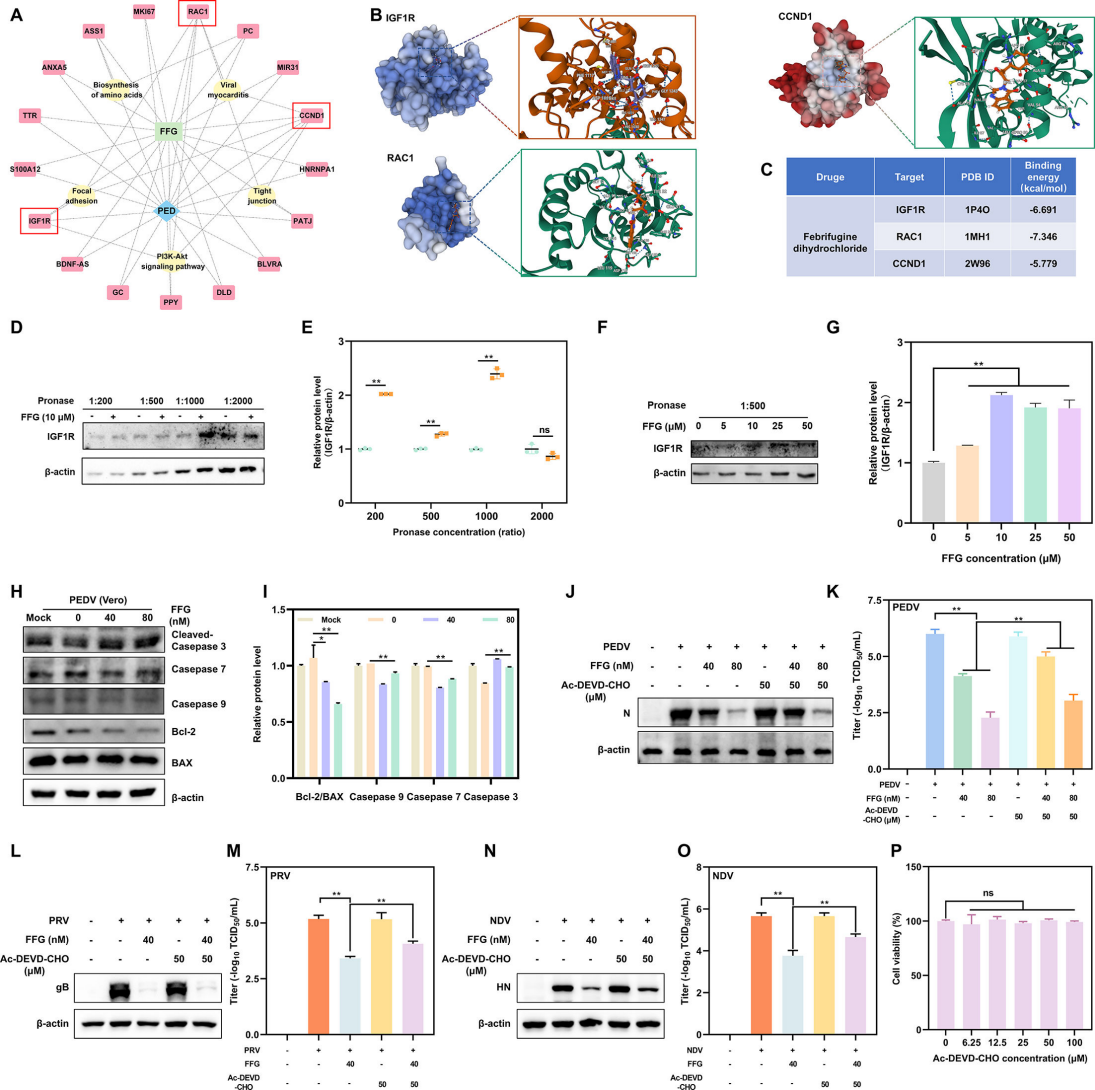
FFG targeted IGF1R to modulate the apoptotic pathway and restrict viral infection by inhibiting the activation of the PI3K/AKT pathway. (**A**) The drug&disease-targets-pathways network analysis. (**B**) Molecular docking simulation of FFG with IGF1R, RAC1, and CCND1. (**C**) Binding energy scores of FFG with the predicted targets. (**D and E**) FFG protected IGF1R against proteolysis. The cell lysates were incubated with FFG (10 μM) and subsequently treated with increasing concentrations of pronase. Western blotting (**D**) and quantification (**E**) were used to assess the stability of IGF1R against proteolysis via DARTS. Western blotting (**F**) and quantification (**G**) were used to assess the stability of IGF1R for cell lysates incubated with increasing concentrations of FFG against proteolysis. (**H and I**) FFG induced apoptosis in PEDV-infected Vero cells. The cells were treated with FFG for 24 hpi, followed by analysis of Cleaved-caspase 3, Caspase-7, Caspase-9, Bcl-2, and BAX expression by Western blotting (**H**) and quantitative densitometric analysis (**I**). (J–O) Inhibition of apoptosis attenuated the antiviral activity of FFG. Vero cells were infected with 0.1 MOI PEDV, 0.1 MOI PRV, or 0.01 MOI NDV and treated with FFG in the presence of 50 μM Ac-DEVD-CHO (Caspase-3 specific inhibitor) for 24 hpi. Viral protein expression (**J, L, N**) and viral titers in culture supernatants (**K, M, O**) were subsequently determined. (**P**) The cell viability of Ac-DEVD-CHO in Vero cells.

To investigate how FFG exerted antiviral activity by modulating the PI3K/AKT pathway, we established the drug&disease-targets-pathways network with common targets of FFG and PED (Fig. 8A). The analysis revealed that FFG might regulate the PI3K/AKT pathway by targeting IGF1R, RAC1, and CCND1. Molecular docking further supported this prediction, showing that FFG stably bound within the ligand-binding pockets of these targets with favorable binding energies (Fig. 8B and C). IGF1R acts as a primary upstream regulator of the PI3K/AKT pathway (30, 31), and our previous research demonstrated that FFG modulated the pathway to exert antiviral effects. Consequently, we hypothesized that FFG directly linked IGF1R to PI3K/AKT pathway regulation. To further determine whether FFG directly interacted with IGF1R, the drug affinity responsive target stability (DARTS) analysis was performed. DARTS is a label-free method based on the principle that small-molecule binding stabilizes target proteins against proteolytic degradation (32). As shown in Fig. 8D and E, FFG strongly altered IGF1R stability under increasing pronase concentrations. Similar results were obtained in DARTS assays using cells treated with increasing concentrations of FFG, in which IGF1R exhibited progressively reduced susceptibility to pronase-mediated proteolysis as FFG concentration increased (Fig. 8F and G).

Given that viruses frequently exploit the PI3K/AKT axis to maintain host cell survival and establish a permissive environment for replication, we next sought to determine which downstream branch of this pathway mediates FFG’s antiviral effects. Among the PI3K/AKT-regulated processes, apoptosis is particularly noteworthy, as many viruses actively suppress programmed cell death to maximize their replication efficiency (33, 34). To determine whether apoptosis serves as the downstream effector through which FFG restricts PEDV infection, we examined apoptotic signaling following FFG treatment. FFG markedly increased apoptotic activity in PEDV-infected cells, as evidenced by increased levels of Cleaved-caspase 3 and decreased levels of Bcl-2/BAX ratio, Caspase 7, and Caspase 9 (Fig. 8H and I). These results indicated that FFG could induce apoptosis during PEDV infection. Consistent with this observation, pharmacological inhibition of apoptosis attenuated the antiviral efficacy of FFG. As shown in Fig. 8J and K, treatment with the Caspase-3 inhibitor Ac-DEVD-CHO significantly suppressed the antiviral activity of FFG, rescuing N protein expression and viral titers, thereby demonstrating that apoptosis was required for FFG-mediated inhibition of PEDV infection. Moreover, Ac-DEVD-CHO similarly rescued FFG’s inhibitory effects against PRV and NDV (Fig. 8L–O). Notably, Ac-DEVD-CHO alone did not affect viral infection and cell viability (Fig. 8P), but specifically counteracted FFG’s antiviral effects, confirming apoptosis as a critical downstream mechanism linking the IGF1R-PI3K/AKT axis to viral restriction.

## DISCUSSION

In recent decades, PEDV has emerged as a major threat to the swine industry, leading to substantial economic losses. Although several effective vaccines are available, their protective efficacy may be compromised by viral mutations, underscoring the urgent need to develop novel agents (1, 35). Numerous studies have reported that *Dichroa febrifuga*-derived quinazolinone alkaloids have a wide range of biological activities, including anticancer, antiparasitic, and therapeutic effects on fibrosis-related and autoimmune diseases (36, 37). However, their antiviral effects have rarely been investigated. Therefore, systematic screening of antiviral agents from *Dichroa febrifuga*-derived quinazolinone alkaloids is potentially valuable. Our research found that the quinazolinone alkaloids exhibited various degrees of activity against PEDV. Among them, febrifugine dihydrochloride (FFG) exhibited submicromolar antiviral efficacy with a favorable selectivity index (SI = 13.4); however, its CC₅₀ of 0.510 μM suggests a relatively low concentration safety margin at the cellular level (Fig. 1). Furthermore, experimental results demonstrated that the antiviral activity of FFG was independent of the infection dose and the subtype of PEDV *in vitro* (Fig. 2).

Considering the diverse biological activities of FFG, we hypothesized that FFG might possess broad-spectrum antiviral properties. Consequently, we systematically evaluated the antiviral potential of FFG against several prevalent and harmful pathogens, including PCV-2, PRV, PRRSV, influenza A virus (H1N1), and NDV. Notably, our study revealed that FFG significantly inhibited various viral infections at a low concentration of 80 nM, highlighting its broad-spectrum antiviral efficacy against both DNA and RNA viruses. Meanwhile, the CC₅₀ values of FFG in the corresponding host cell lines for tested viruses indicated that its antiviral activity was achieved at concentrations that did not induce overt cytotoxicity (Fig. 3). An ideal antiviral agent should effectively target the viral replication stage, as this stage is critical for the amplification of the viral genome. The replication stage represents a crucial juncture in the viral life cycle, during which the virus synthesizes its genetic material and produces progeny virions. Numerous studies have underscored the significance of targeting viral replication complexes. For example, the RNA-dependent RNA polymerase (RdRp) of positive-sense RNA viruses serves as a crucial enzyme in the replication process and has been recognized as a promising target for antiviral therapeutics (38, 39). Building on this premise, we investigated the mechanism underlying FFG-mediated inhibition of PEDV by administering FFG at distinct stages of the viral life cycle. Our results demonstrated that FFG’s anti-PEDV activity was achieved through targeted inhibition of PEDV replication, without impacting other stages (Fig. 4 and 5). These findings suggest that FFG exerts its antiviral effects during the replication stage by disrupting signaling pathways essential for viral genome amplification and protein synthesis.

Through network pharmacology analysis, several critical signaling pathways have been identified as potentially involved in the antiviral activity of FFG, with the PI3K/AKT pathway emerging as a significant regulatory hub associated with multiple target genes (Fig. 6). The enrichment of PI3K/AKT-related targets in the network analysis strongly suggests that FFG may exert its antiviral effects by modulating this host signaling cascade. It is well established that the PI3K/AKT pathway plays a crucial role in coordinating cell survival, apoptosis, metabolism, and immune responses, and many viruses exploit this pathway to create a favorable environment for their replication. For instance, the V protein of canine distemper virus triggers autophagy through the PI3K/AKT pathway to enhance viral replication (40). Similarly, mammalian DNA viruses have evolved diverse strategies to activate the PI3K/AKT pathway to benefit from AKT-mediated signaling and promote viral proliferation (41). Meanwhile, previous research has shown that the activation of PI3K/AKT by viral epithelial proteins present during herpesvirus entry is critical for viral infection and optimization of replication (42). To validate the predictions derived from network pharmacology, we conducted experimental investigations to assess the impact of FFG on the PI3K/AKT signaling pathway in virus-infected cells. Our results revealed that FFG significantly inhibited virus-induced activation of the PI3K/AKT pathway during infections with PEDV, H1N1, PRV, and PRRSV, indicating that its antiviral efficacy is closely linked to the suppression of PI3K/AKT signaling (Fig. 7). Similarly, Dunn et al. reported that AKT-IV, an AKT inhibitor, broadly inhibited viral replication (43). Zhang et al. also systematically elucidated that the PI3K/AKT signaling cascade plays a crucial role in HPV-induced carcinogenesis through multiple cellular and molecular events (44). Additionally, AR-12, a derivative of celecoxib, has been shown to inhibit dengue virus infection via the PI3/AKT pathway (45). Collectively, these studies underscore the PI3K/AKT pathway as a critical host antiviral target and support the conclusion that FFG exerts broad-spectrum antiviral effects through modulation of this signaling axis at nanomolar concentrations.

Our mechanistic investigation revealed that among the various potential upstream regulators predicted by network pharmacology, IGF1R emerged as a major candidate target. Numerous studies have established IGF1R as a crucial activator of the PI3K/AKT pathway (46, 47). Consistent with this prediction, our experimental results demonstrated that FFG directly interacts with IGF1R, thereby inhibiting virus-induced PI3K/AKT activation. This finding firmly positions IGF1R as the primary molecular target through which FFG exerts its antiviral effects. Furthermore, research has indicated that rotaviruses initiate autophagy by targeting IGF1R to activate the PI3K/AKT pathway, thus fostering an environment favorable for self-replication (47). Given that PI3K/AKT signaling serves as a central upstream regulator of apoptosis, we further explored whether the downstream apoptotic pathway contributes to FFG’s antiviral activity. Fan et al. reported that NDV activated the PI3K/AKT signaling pathway degradation to delay cell apoptosis to facilitate its replication (33). In contrast, our study revealed that FFG treatment induced the apoptotic process. Importantly, the inhibition of apoptosis using Ac-DEVD-CHO significantly attenuated FFG’s antiviral efficacy, confirming apoptosis as a critical downstream effector in FFG’s antiviral mechanism (Fig. 8). Previous studies have demonstrated that the antiparasitic activity of FFG is primarily attributed to its direct inhibition of host prolyl-tRNA synthetase, thereby disrupting protein synthesis (37, 48). In contrast, our study reveals that FFG exerts its antiviral activity through modulation of the IGF1R-PI3K/AKT-apoptosis axis. Although its antiparasitic and antiviral mechanisms are distinct, both underscore FFG’s ability to interfere with critical host physiological processes to restrict pathogen survival and replication, further highlighting its potential as a therapeutic agent against multiple pathogens.

**Fig 9:**
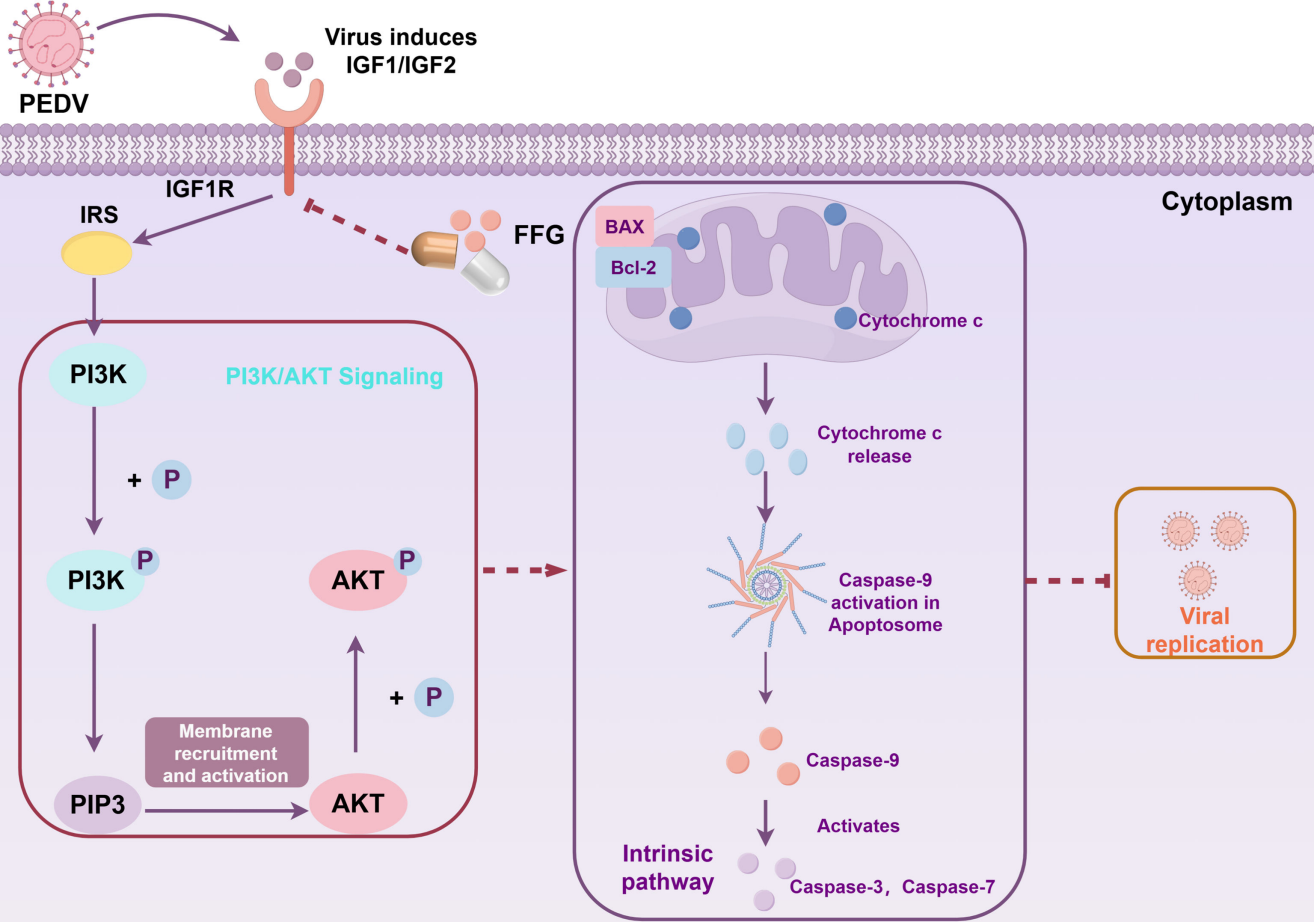
Schematic diagram of FFG’s mechanism against PEDV.

In this study, we screened a series of quinoline alkaloids derived from *Dichroa febrifuga* as antiviral agents against PEDV. FFG emerged as a compound with exceptional antiviral activity against multiple viruses, specifically exerting its effects during the replication stage of the viral life cycle. Mechanistic analyses elucidated that FFG exerts its antiviral activity via a novel IGF1R-PI3K/AKT-apoptosis axis by targeting IGF1R (Fig. 9). This study not only identifies FFG as a promising novel antiviral candidate that targets the host but also serves as a reference for the development of therapeutic agents against viral infections.

## MATERIALS AND METHODS

### Cell culture and virus preservation

PEDV/HLJBY was isolated from the intestinal contents of a diarrheic piglet in Heilongjiang Province, and PEDV reference strain CV777 was purchased from the China Institute of Veterinary Drug Control. PRRSV, PRV (XJ5 strain), PCV-2, influenza A virus H1N1 (PR8 strain), and NDV were preserved in our laboratory. PEDV and PRRSV were propagated in Vero and Marc-145 cells, respectively; PRV and PCV-2 were propagated in PK-15 B6 cells; H1N1 and NDV were propagated in 10-day-old SPF embryonated chicken eggs. Viral titers were determined by the TCID₅₀ method or HA assay, depending on viral type. Laboratory-preserved porcine alveolar macrophages (PAMs) were isolated from the lungs of PRRSV-negative piglets via lung lavage. All cells were cultured in Dulbecco’s modified Eagle’s medium (DMEM, Sigma) with 1% penicillin-streptomycin and fetal bovine serum (FBS, Lonsera) at 37°C with 5% CO_2_.

### Chemicals, reagents, and antibodies

The febrifugine (catalog number HY-N2384), febrifugine dihydrochloride (catalog number HY-N2384A), halofuginone (catalog number HY-N1584), halofuginone hydrobromide (catalog number HY-N1584A), isofebrifugine (catalog number HY-N5029), and Ac-DEVD-CHO (catalog number HY-P1001) were purchased from MCE (NJ, USA). LY294002 (catalog number S1105) was purchased from Selleck (TX, USA). The citric acid solution, containing 40 mM citric acid, 10 mM KCl, and 135 mM NaCl, was adjusted to pH 3.0 and used to remove non-internalized viral particles. Monoclonal antibodies against the proteins of PEDV N, PRV gB, PCV-2 Cap, H1N1 NP, PRRSV N, and NDV HN were developed in our laboratory. These monoclonal antibodies were diluted 1:1,000 for Western blotting. β-actin monoclonal antibody (1:1,000, catalog number HC201-01), PerfectStart II Probe qPCR SuperMix Kit (catalog number AQ711-01), and EasyScript One-Step gDNA Removal and cDNA Synthesis SuperMix Kits (catalog number AE311-02) were purchased from *TRANS* (Beijing, China). DAPI (catalog number C1005) and CCK-8 reagent (catalog number C0038) were purchased from Beyotime Biotechnology (Shanghai, China). PI3 Kinase p110β polyclonal antibody (1:1,000, catalog number 3011) and AKT polyclonal antibody (1:1,000, catalog number 9272) were purchased from Cell Signaling Technology (MA, USA). The p-AKT polyclonal antibody (1:1,000, catalog number WLP001) and Cleaved-caspase 3 polyclonal antibody (1:1,000, catalog number WL02117) were purchased from Wanlei Biological Technology Co., Ltd (Shenyang, China). The p-PI3 Kinase polyclonal antibody (1:1,000 catalog number T40116) was purchased from Abmart Shanghai Co., Ltd (Shanghai, China). BAX polyclonal antibody (1:1,000, catalog number 50599-2-Ig), Bcl-2 polyclonal antibody (1:1,000, catalog number 12789-1-AP), Caspase 7 polyclonal antibody (1:1,000, catalog number 27155-1-AP), and Caspase 9/P35 polyclonal antibody (1:1,000, catalog number 10380-1-AP) were purchased from Proteintech (Inc., USA). HRP-labeled goat anti-mouse IgG (H+L) (1:5,000, catalog number A0216) and HRP-labeled goat anti-rabbit IgG (H+L) (1:5,000, catalog number A0208) were purchased from Beyotime Biotechnology. FITC-labeled rabbit anti-pig IgG (1:1,000, catalog number F1638) was purchased from Sigma.

### Cytotoxicity assay of drugs

The cytotoxicity of febrifugine, febrifugine dihydrochloride, halofuginone, halofuginone hydrobromide, and isofebrifugine was evaluated using CCK-8 assays. Briefly, multiple cell lines and PAMs were seeded into 96-well plates and incubated for 24 h. Upon reaching approximately 80% confluence, the culture medium was replaced with 2% FBS DMEM and serial twofold dilutions of each compound. After 24 h of incubation, 10 μL of CCK-8 reagent was added to each well, followed by incubation for an additional 1 h. Absorbance was measured at 450 nm using a microplate reader, and CC₅₀ (with 95% confidence intervals) values were calculated using GraphPad Prism.

### Infectivity assay

The multiple cell lines were seeded in 12-well plates and incubated until reaching 70%–80% confluence. The culture medium was removed, and cells were washed three times with PBS. Subsequently, 1 mL of serum-free DMEM containing FFG at final concentrations of 10, 20, 40, or 80 nM was added to each well, followed by incubation for 1 h at 37°C. Cells were then infected with different viruses in the presence of FFG at the corresponding concentrations, including PEDV (MOI = 0.1), PRV (MOI = 0.1), PRRSV (MOI = 0.1), PCV-2 (MOI = 0.5), H1N1 (MOI = 1), and NDV (MOI = 0.01). After 1 h at 37°C, the inoculum was removed and replaced with 1 mL of 2% FBS DMEM containing the corresponding concentration of FFG. Infected cells were maintained under these conditions for 24 hpi, after which cell lysates and culture supernatants were collected for Western blotting and TCID₅₀ assays. Parallel samples were fixed for an indirect immunofluorescence assay (IFA) to assess internalized virus.

### Antiviral activity of FFG at different stages of PEDV life cycle

The drug-addition assay was performed as previously described to determine the antiviral mode of FFG (49). Briefly, for the adsorption assay, Vero cells were treated with varying concentrations of FFG, followed by infection with 0.1 MOI PEDV/HLJBY at 4°C for 1 h. After incubation, cells were washed three times with PBS and incubated with 2% FBS DMEM at 37°C for 24 hpi. For the entry assay, Vero cells were infected with 0.1 MOI PEDV/HLJBY at 4°C for 1 h without FFG. After washing three times with cold PBS, cells were treated with 2% FBS DMEM containing various concentrations of FFG for 1 h at 37°C, followed by washing with citric acid solution and PBS. The cells were then incubated with 2% FBS DMEM without FFG for 24 hpi. For the replication assay, Vero cells were infected with 1 MOI PEDV/HLJBY for 1 h, washed with PBS, and then incubated with 2% FBS DMEM containing different concentrations of FFG. Cells were harvested at 4 and 6 hpi for subsequent analyses. Western blotting, IFA, qRT-PCR, and TCID₅₀ assays were conducted to assess viral infection level.

### Western blotting and calculation of EC_50_

The cells were lysed on ice using pre-cooled RIPA buffer supplemented with protease inhibitors. Equal amounts of whole-cell protein were resolved on SDS-PAGE gels and subsequently transferred onto NC membranes via wet transfer at a constant current of 200 mA for 100 min at 4°C. Membranes were blocked with 5% skim milk at room temperature for 2 h, followed by incubation with primary antibodies overnight at 4°C and HRP-conjugated secondary antibodies for 2 h at room temperature. Signals were visualized using ECL, and images were analyzed using ImageJ. Dose-response curves were generated using GraphPad Prism, with FFG concentration (log_2_-transformed) plotted on the X-axis and percentage inhibition on the Y-axis. EC₅₀ values (with 95% confidence intervals) were obtained directly from the fitted model.

### Virus titer assays

The cell lines used for viral propagation were seeded into 96-well plates and cultured until reaching 70%–80% confluence. The virus samples to be tested were serially diluted in 10-fold gradients and inoculated onto the cells, followed by incubation for 1.5 h. After incubation, the cells were washed three times with PBS before the addition of 2% FBS DMEM. After 72 h of incubation, the cytopathic effect (CPE) was examined under an inverted microscope, and the 50% tissue culture infectious dose (TCID₅₀) values of the samples were calculated using the Reed-Muench method.

### qRT-PCR

Reverse transcription was performed using the EasyScript One-Step gDNA Removal and cDNA Synthesis SuperMix kit to convert RNA to cDNA. The qPCR mixture was then prepared according to the Universal qPCR Master Mix kit. The reaction conditions were as follows: pre-denaturation at 95°C for 30 s, denaturation at 95°C for 10 s, and annealing at 60°C for 30 s for a total of 45 cycles. Primer sequences: PEDV N-F: GAATTCCCAAGGGCGAAAAT, PEDV N-R: TTTTCGACAAATTCCGCATCT, N probe: 5′-FAM-CGTAGCAGCTTGCTTCGGACCCA-BHQa-3'.

### Indirect immunofluorescence assay

The cells were fixed at 37°C for 30 min with 4% paraformaldehyde, incubated with 0.1% Triton X-100 for 10 min, and then blocked overnight at 4°C with 5% BSA. After washing with PBS, cells were incubated at 37°C for 1 h with anti-PEDV N or PCV-2 Cap antibodies, followed by incubation with FITC-labeled goat anti-mouse IgG antibody at 37°C for 1 h. Finally, cells were stained with DAPI for 7 min and observed under a fluorescence microscope.

### Target prediction and analysis of FFG and PED

The potential targets of PED were obtained from GeneCards (https:// www.genecards.org/). Subsequently, the potential targets of FFG were predicted using GeneCards, PharmMapper (http://lilab-ecust.cn/pharmma pper/index.html), and STITCH (http://stitch.embl.de/) databases. The targets were standardized using the UniProt database (https://www.uniprot.org/) and converted to gene names. Finally, FFG targets were gathered after removing duplicates.

### PPI, GO, and KEGG enrichment analysis, and molecular docking

The compound targets of FFG and PED were identified using a bioinformatics platform (https://www.bio informatics.com.cn/) to generate Venn diagrams. Overlapping targets were submitted to the STRING database (https://string-db.org/) settings to construct PPI networks. GO enrichment and KEGG pathway analyses were performed using the DAVID database with the appropriate species selected. The top 10 pathways ranked by *P* values were visualized using the Bioinformatics platform. Finally, the drug-target-disease visualization network and drug-target-disease-pathway networks were constructed in Cytoscape. In addition, AutoDock Vina, an *in silico* protein-ligand docking program, was used to assess the binding affinities and interaction patterns between FFG and its potential targets (50). The molecular structure of FFG was retrieved from PubChem (https://pubchem.ncbi.nlm.nih.gov/). The 3D coordinates of RAC1, IGF1R, and CCND1 were downloaded from the PDB (http://www.rcsb.org/pdb/home/home.do). For docking analysis, all protein and molecular files were converted into PDBQT format, with all water molecules excluded, and polar hydrogen atoms were added. The grid box was centered to cover the domain of each protein and accommodate free molecular movement. The grid box was set to 30 Å × 30 Å × 30 Å, and the grid point distance was 0.05 nm. Molecular docking studies were performed by AutoDock Vina.

### Drug affinity responsive target stability assay

The drug affinity responsive target stability (DARTS) assay was performed according to previously reported methods with minor modifications (32). Briefly, Vero cells in culture dishes were scraped, centrifuged, and resuspended in pre-chilled PBS. The resuspension was subjected to three freeze-thaw cycles using liquid nitrogen. After centrifugation at 12,000 rpm for 20 min, the supernatant was collected, and protein concentrations were determined. The cell lysates were then incubated at room temperature with DMSO or varying concentrations of FFG for 2 h. Following incubation, trypsin was added at different ratios and incubated for 30 min, after which a protease inhibitor cocktail was added to terminate proteolysis. Subsequently, 4 × SDS-PAGE loading buffer was added, and the samples were heated at 96°C for 10 min. The processed samples were then subjected to Western blotting.

### Statistical analysis

Each assay was independently replicated a minimum of three times, with results presented as the mean ± SD. Statistical analyses were conducted utilizing GraphPad Prism (San Diego, CA, USA). Differences between groups were assessed using one-way analysis of variance (ANOVA) and t-tests. Statistical significance is indicated as follows: *P < 0.05, **P < 0.01, and ns indicates not significant.

## Data Availability

All data generated or analyzed during this study are included in the paper. The databases and software used in this study are publicly available and cited in Materials and Methods.

## SUPPLEMENTAL MATERIAL

Fig. S1 jvi.00117-26-s0001.docx Schematic overview of the study.

ASM does not own the copyrights to Supplemental Material that may be linked to, or accessed through, an article. The authors have granted ASM a non-exclusive, world-wide license to publish the Supplemental Material files. Please contact the corresponding author directly for reuse.
