# Supplementary material for: Febrifugine dihydrochloride restricts porcine epidemic diarrhea virus replication by modulating the IGF1R-driven PI3K/AKT-apoptosis axis
Source: J Virol. 2026 Apr 15;100(5):e00117-26. doi: 10.1128/jvi.00117-26 (PMC13185639; doi:10.1128/jvi.00117-26)
Supplement: Fig. S1 — Schematic overview of the study. [file jvi.00117-26-s0001.docx]

Supporting Information

**Febrifugine dihydrochloride Restricts Porcine Epidemic Diarrhea Virus Replication by Modulating the IGF1R-Driven PI3K/AKT-Apoptosis Axis**

Ping Yan *^a,b,c^, K.Y.A.W.Z.I.N.OO *^a,b,c^, Xiaobing Wang *^a,b,c^, Nan Li ^a,b,c^, Yulan Xu ^a,b,c^, Song Gao ^# a,b,c^, Changchao Huan ^# a,b,c^.

^a^ Institute of Agricultural Science and Technology Development, College of Veterinary Medicine, Yangzhou University, Yangzhou, China

^b^ Jiangsu Co-Innovation Center for Prevention and Control of Important Animal Infectious Diseases and Zoonoses, Yangzhou, China

^c^ Key Laboratory of Avian Bioproduct Development, Ministry of Agriculture and Rural Affairs, Yangzhou, China





**FIG S1** Schematic overview of the study.
